# No evidence for association between late pregnancy maternal cortisol and gray matter volume in a healthy community sample of young adolescents

**DOI:** 10.3389/fnins.2022.893847

**Published:** 2022-08-31

**Authors:** Anna Tyborowska, Katharina Gruber, Roseriet Beijers, Simone Kühn, Karin Roelofs, Carolina de Weerth

**Affiliations:** ^1^Behavioural Science Institute, Radboud University, Nijmegen, Netherlands; ^2^Donders Centre for Cognitive Neuroimaging, Donders Institute for Brain, Cognition and Behaviour, Radboud University, Nijmegen, Netherlands; ^3^Department of Psychiatry, Donders Institute for Brain, Cognition and Behaviour, Radboud University Nijmegen Medical Centre, Nijmegen, Netherlands; ^4^Department of Cognitive Neuroscience, Donders Institute for Brain, Cognition and Behaviour, Radboud University Medical Center, Nijmegen, Netherlands; ^5^Lise Meitner Group for Environmental Neuroscience, Max Planck Institute for Human Development, Berlin, Germany; ^6^Clinic and Policlinic for Psychiatry and Psychotherapy, University Clinic Hamburg-Eppendorf, Hamburg, Germany

**Keywords:** glucocorticoids, prenatal, fetal, brain, GMV, adolescence

## Abstract

A compelling amount of animal and human research has shown that perceived maternal stress during pregnancy can affect the neurodevelopment of the offspring. Prenatal maternal cortisol is frequently proposed as the biological key mechanism underlying this link; however, literature that investigates the effects of prenatal cortisol on subsequent neurodevelopment in humans is scarce. By using longitudinal data from a relatively large community sample of mother–child dyads (*N* = 73), this pre-registered study prospectively examined the role of maternal prenatal cortisol concentrations on subsequent individual differences in gray matter volume (GMV) and hippocampal subfield volumes at the onset of puberty of the offspring (12 years of age). Two markers of cortisol, that is, evening cortisol and circadian decline over the day, were used as indicators of maternal physiological stress during the last trimester of pregnancy. The results indicate that prenatal maternal cortisol levels were not associated with GMV or hippocampal subfield volumes of the children. These findings suggest that late pregnancy maternal cortisol may not be related to the structural development of the offspring’s brain, at least not in healthy community samples and at the onset of puberty. When examining the influence of prenatal stress on offspring neurodevelopment, future investigations should delineate gestational timing effects of the cortisol exposure, cortisol assessment method, and impact of additional biomarkers, as these were not investigated in this study.

## Introduction

During the gestational period, the fetal brain undergoes enormous maturational processes, making it highly susceptible to environmental factors. Maternal stress during pregnancy, as one of these environmental factors, has been linked to long-lasting alterations in offspring behavior and brain structure (for reviews see Franke et al., 2020; van den Bergh et al., 2020). Most findings, however, stem from self-reported maternal stress measures (Buss et al., 2010; Favaro et al., 2015; Acosta et al., 2019; Jones et al., 2019; Marečková et al., 2019), while much less is known how prenatal exposure to the stress hormone cortisol is associated with offspring brain outcomes. In this prospective longitudinal study, we investigated individual differences in offspring brain structure associated with prenatal maternal cortisol concentrations. Importantly, we focused on two critical developmental periods: (1) late pregnancy, when maternal cortisol levels peak and the fetal brain undergoes a sensitive period of proliferation (Andersen, 2003), and (2) onset of puberty, a second period of neural reorganization, particularly in areas involved in social-emotional processing (Blakemore, 2008; Schulz and Sisk, 2016).

Cortisol (glucocorticoids in animals), the end product of the hypothalamic–pituitary–adrenal (HPA) axis, is one of the major players in the body’s neuroendocrine response to psychological or physical stressors (Sapolsky et al., 2000). As stressors increase, cortisol concentrations can rise and become chronically heightened (Gunnar and Quevedo, 2007). As such, cortisol is seen as a biomarker of stress (Cottrell and Seckl, 2009). During the normal course of gestation, maternal cortisol also rises 3- to 5-fold, reaching the highest concentrations in the late gestational period (Sandman et al., 2006). Subsequently, elevated cortisol concentrations of the mother can increase fetal cortisol levels by stimulating the release of placental corticotrophin-releasing hormone (CRH), which in turn stimulates the fetal HPA axis (Majzoub and Karalis, 1999), or by crossing the placenta and directly entering fetal circulation (Gitau et al., 1998). Though the fetus is partly protected from elevated maternal cortisol through the placental enzyme 11β-hydroxysteroid dehydrogenase type 2 (11β-HSD2), the activity of 11β-HSD2 declines across pregnancy, potentially allowing higher concentrations of maternal cortisol to reach the fetal brain in late pregnancy (Murphy and Clifton, 2003).

The hippocampus is one of the target areas of prenatal stress research, given its pivotal role in memory formation and emotion regulation and its implication in a variety of psychiatric disorders and brain plasticity (Phelps, 2004; Sala et al., 2004; Knierim, 2015). Together with the prefrontal cortex and the amygdala, the hippocampus contains a particularly high concentration of glucocorticoid receptors, making it highly susceptible to the effect of stress hormones (Sapolsky et al., 2000). Particularly during puberty, the long-lasting effects of earlier stress exposure become apparent, especially in these stress-sensitive areas (Lupien et al., 2009). This is due to ongoing structural modifications, characterized by synaptic pruning of connections and thought to underlie normative reductions of gray matter volume during adolescence (GMV; Giedd et al., 1999). Increased reductions in GMV during this time have particularly been ascribed to stress experienced early in life (Carrion and Wong, 2012; Cohen et al., 2013). These effects are thought to reflect either toxic effects of glucocorticoids causing dendritic spine loss or cell death (Sapolsky et al., 1986; Lupien et al., 2009), or accelerated maturation of neural circuits due to an evolutionary prioritization of maturational processes (Callaghan and Tottenham, 2016). However, particularly with respect to the hippocampus and amygdala, findings in adolescents remain inconsistent, with some cross-sectional or between-group studies reporting stress-related GMV differences or null effects (De Bellis et al., 2001; Tupler and De Bellis, 2006; Mehta et al., 2009; Tottenham et al., 2010). Conflicting findings may be related to measurement period and the type of stressor that is measured (Teicher et al., 2016). In line with this, early amygdala GMV enlargement is suggested to occur in response to adversity, but is later followed by premature volume reduction (Tottenham and Sheridan, 2010).

A handful of studies in humans have related prenatal stress to offspring structural neural alterations. Self-reported prenatal maternal stress was associated with increased fetal cortical gyrification (Wu et al., 2020) and with a slower rate of postpartum hippocampal growth in 6-month-old infants (Qiu et al., 2013). During middle childhood, self-reported prenatal stress was related to lower cortical thickness and lower GMV in the frontal, prefrontal, and temporal lobes (Buss et al., 2010; Davis et al., 2020), whereas in adolescents (aged 11–14 years), it was associated with higher posterior parietal cortex density (McQuaid et al., 2019). Larger amygdala GMV associated with prenatal maternal stress was also reported in children and female, but not male, adolescents (Wen et al., 2017; Acosta et al., 2019; Jones et al., 2019). A few studies have also looked at relationships between prenatal stress and GMV in young adulthood. For example, negative life events experienced by the mother during pregnancy have been linked to lower GMV in the adult offspring’s dorsolateral frontal cortex, anterior cingulate cortex, and precuneus (Marečková et al., 2019), and specifically in the amygdalar accessory basal and cortical nuclei volumes (Mareckova et al., 2022). Taken together, these studies point at potential influences of maternal stress during pregnancy on the development of offspring brain morphometry.

Even fewer studies have examined associations of prenatal maternal cortisol concentrations with the child’s structural development. Cortisol is likely a unique predictor of neural development given the weak relation between prenatal maternal self-reported psychosocial stress measures and prenatal maternal cortisol measures (Beijers et al., 2010, 2014). Fetal brain development during late pregnancy is characterized by immense morphological reorganization and programmed cell death (Andersen, 2003) with glucocorticoids playing a crucial role in normal brain development (Davis and Sandman, 2010). Both elevated and suppressed levels have been hypothesized to induce neurotoxicity with adverse and long-lasting effects on brain structure (Uno et al., 1990, 1994; Lupien et al., 2009). Elevated prenatal maternal cortisol levels have been related to enlarged amygdala, but not hippocampus volume, and more affective symptoms in girls, but not boys. These effects were apparent for cortisol levels during early, but not late pregnancy (Buss et al., 2012). However, in newborns, amygdala architecture and connectivity, but not volume, were associated with prenatal maternal cortisol (Stoye et al., 2020). During middle childhood, segregation of structural neural networks has been linked to prenatal maternal cortisol, but only in girls (Kim et al., 2017). Others have reported higher cortical thickness in frontal regions and enhanced cognitive performance in 6- to 9-year-old children of mothers with elevated cortisol concentrations during the third trimester (Davis et al., 2017). These findings suggest that elevated prenatal maternal cortisol may exert lasting effects on brain development, although the precise nature of these effects may depend on other factors, such as gestational age and offspring sex.

Animal studies provide additional evidence that aberrant cortisol exposure can have detrimental consequences for the developing brain, especially the hippocampus. Lower hippocampal volume and inhibited neurogenesis in the dentate gyrus were found in 2- to 3-year-old rhesus monkeys exposed to stress in early and late pregnancy (Coe et al., 2003). Similar findings were reported as a result of glucocorticoid treatment in late pregnancy, which resulted in lower hippocampal volume in rhesus monkeys at 20 months of age (Uno et al., 1990), as well as differences in the development of the cortical layer and diminished brain growth in rodents (Velazquez and Romano, 1987).

In sum, maternal cortisol is often proposed as the major physiological mechanism underlying the relation between prenatal stress and altered childhood outcomes (Cottrell and Seckl, 2009). However, studies investigating brain correlates of prenatal maternal cortisol are scarce. The aim of this study was two-fold. First, we prospectively aimed to examine whole-brain associations between late-pregnancy maternal cortisol levels and subsequent child GMV, by using data from a healthy community sample. To our knowledge, longitudinal human research has investigated neural associations with prenatal cortisol up to 6–9 years of age (Buss et al., 2012; Davis et al., 2017). We aimed to extend these findings by investigating whether incubated effects of maternal cortisol manifest on brain structure in early adolescence (age 12), a sensitive period of social-emotional development (Blakemore, 2008; Crone and Dahl, 2012). Second, we intended to explore these effects in separate hippocampal subfields. Although the hippocampus is often treated as a homogeneous structure (e.g., total volume), previous research suggests that various hippocampal subfields may be differentially affected by early life stress (Teicher et al., 2012). We expect that regions with a high distribution of glucocorticoid receptors will be most susceptible to prenatal cortisol concentrations, namely the prefrontal cortex, amygdala, and hippocampus, with differential subfield associations. We will also apply whole-brain statistical inferences.

## Materials and methods

### Participants

For this magnetic resonance imaging (MRI) study, we approached children and their parents from the “Basale Invloeden op de Baby Ontwikkeling” (BIBO; English: “Basal Influences on the Baby’s Development”) study. The BIBO study is an ongoing longitudinal cohort study, which originally started with 193 Dutch mother–child community dyads and aims to investigate the influences of early environmental factors and individual characteristics on child development. Inclusion criteria were an uncomplicated and singleton pregnancy, no drug use during pregnancy, no current physical health problems (e.g., diabetes, heart disease) and mental health problems (e.g., major depression), a term delivery (≥37 weeks), and a normal 5-min infant Apgar score (≥7). For more information on participant recruitment and inclusion criteria, see also previous studies (Beijers et al., 2010, 2017; Simons et al., 2019). See Table 1 for the demographic characteristics of the sample.

**TABLE 1:** Descriptive statistics of the mother and child.

	Mean ± SD	Min/max
Age at MRI scan (years)	Boys: 12.78 ± 0.27	12.22/13.47
	Girls: 12.77 ± 0.27	12.30/13.50
Birth weight (g)	3662 ± 453	2708/4700
Gestational length (days)	282 ± 7.2	266/295
5 min Apgar score	9.5 ± 0.67	7/10
Unassisted deliveries	84.9%	
First born	39.7%	
Number of siblings	0.82 ± 0.74	0/2
Mother’s age at child’s birth (years)	33.1 ± 3.67	23.6/42
Maternal higher education (College or University)	80.8%	
Smoking during pregnancy	1.4%	
Alcohol during pregnancy	12.3%	

Boys and girls in the sample did not differ in age [t(69.95) = −0.17, p > 0.05].

Anatomical MRI scans were acquired for 97 children as part of a larger data collection wave for the BIBO study at the age of 12 years (*M* = 12.78, *SD* = 0.27). Children were excluded from MRI assessment if they had a severe physical or mental disability, as well as standard counter-indications for MRI. All children had normal or corrected-to-normal vision and were familiarized with the scanning environment prior to the actual MRI session (simulation scanner without the presence of a magnetic field). Structural MRI data of two children were excluded because of poor data quality (for exclusion criteria of MRI data see Section “Magnetic resonance imaging preprocessing and analysis”). Out of the children with acceptable MRI data, prenatal maternal cortisol data were available for 74 mother–child dyads. Cortisol data of one mother were indicated as an outlier and excluded from further analysis. This resulted in a final sample of 73 mother–child dyads (39 boys). Previous research in this sample has shown that associations between measures of prenatal stress and infant behavior and symptoms range between an effect size (f^2^) of .1 and .2 (Beijers et al., 2010; Tollenaar et al., 2011). We based our *a priori* effect size on a medium effect of f^2^ = 0.15 at 80% power, resulting in a minimum of 67 participants to test the effect of two predictors. The study was approved by the local ethics committee (CMO regio Arnhem-Nijmegen), and informed consent was obtained from the children and both parents prior to participation. The ethical committee of the Faculty of Social Sciences of Radboud University approved the original study (ECG300107).

### Maternal prenatal cortisol concentrations

During the last trimester of pregnancy (*M* = 37 weeks, 0.8 days; *SD* = 9.4 days), mothers collected salivary samples in 25-ml containers with screw caps. Sampling took place on two consecutive days at five time points to determine fluctuations in cortisol concentrations throughout the day: at wakening, 30 min after wakening, at 12:00, at 16:00, and at 21:00 h (Table 2). The mother was instructed to fill out a form indicating exact times when each sample was collected (even if this did not happen at the planned time). Only the following collection time ranges were used to qualify a sample as acceptable for analysis per time point: (1) between 6:00 and 10:00 and within 15 min after awakening, (2) between 25 and 35 min after awakening, (3) between 11:30 and 13:30, (4) between 15:30 and 17:30, and (5) between 20:00 and 23:00. The samples collected outside of these time ranges were excluded from analyses. In addition, cortisol samples were excluded if they were collected during/after the day of delivery, if the participant reported being sick at the time of sample collection or used medication that could lead to cortisol elevation. The collected samples were stored in a −20°C freezer until analysis. Cortisol concentrations were measured using an in-house competitive radioimmunoassay (Laboratory of Endocrinology, University Medical Center Utrecht, Utrecht, Netherlands). Because of strong inter-correlations of cortisol concentrations between the two days (*r* ranging from 0.53 to 0.68, all *p*-values < 0.01; MRI sample (*n* = 73): *r* ranging from 0.46 to 0.63, all *p*-values <0.001), a mean score over a two-day period was calculated for each sampling time (Table 3). A standardized score of evening cortisol (21:00) and cortisol decline (waking minus evening) were used as variables for prenatal cortisol in the analysis. Previous work using the same sample found that these measures were highly correlated with the cortisol measures—area under the curve (AUCg; *r* = 0.84, *p* < 0.01) and morning cortisol (*r* = 0.85, *p* < 0.01), but not with each other (*r* = 0.11, *p* = 0.17)—making them two strong independent predictors of prenatal cortisol (Beijers et al., 2010; Tollenaar et al., 2011; Simons et al., 2015, 2019; Zijlmans et al., 2017). Measures of evening cortisol and cortisol decline were not related to psychosocial measures of maternal prenatal stress (Beijers et al., 2010). In addition, an exploratory analysis was carried out using the cortisol awakening response (CAR). The CAR is the temporary increase in cortisol levels, which peaks 30 min after awakening and can also be considered an indicator of HPA axis functioning during pregnancy (de Weerth and Buitelaar, 2005a). A standardized mean score of CAR (30 min after wakening minus waking cortisol) over the two-day period was used as a variable in the analysis.

**TABLE 2:** Descriptive statistics of prenatal maternal cortisol levels.

	Boys		Girls	
		
	Mean ± SD	Min/max	Mean ± SD	Min/max
**Day 1 Raw maternal cortisol (nmol/l)**				
Wakening	14.78 ± 4.40	7.80/27.00	16.69 ± 4.67	8.90/29.00
+30 min	20.93 ± 6.69	7.40/39.00	21.11 ± 5.47	11.90/34.00
12:00	15.78 ± 3.38	6.40/22.00	15.68 ± 3.87	9.70/23.00
16:00	12.19 ± 2.85	7.30/17.80	12.63 ± 3.28	5.80/19.30
Evening	9.28 ± 2.82	4.70/18.80	9.91 ± 2.64	3.00/18.10
**Day 2 Raw maternal cortisol (nmol/l)**				
Wakening	16.32 ± 4.18	9.50/25.00	17.68 ± 5.54	7.80/31.00
+30 min	21.93 ± 5.84	9.30/36.00	21.57 ± 6.26	11.70/36.00
12:00	15.66 ± 3.33	8.90/21.00	15.22 ± 3.57	8.00/24.00
16:00	12.27 ± 3.37	5.90/21.00	13.64 ± 2.92	8.20/19.40
Evening	9.32 ± 2.10	4.90/13.50	10.23 ± 2.97	3.60/18.60
**Day 1 & 2 Raw maternal cortisol (nmol/l)**				
Wakening	15.41 ± 3.91	7.80/24.00	16.84 ± 4.71	8.90/29.50
+30 min	21.12 ± 6.11	8.90/35.00	21.25 ± 4.94	11.70/31.50
12:00	15.77 ± 3.07	9.10/22.00	15.35 ± 3.60	8.00/23.50
16:00	12.21 ± 2.66	6.90/18.80	13.10 ± 2.63	8.30/19.00
Evening*	9.39 ± 2.27	5.50/14.80	9.96 ± 2.50	4.60/16.90
Decline*	6.06 ± 4.42	-2.50/16.00	6.91 ± 4.21	-1.40/16.70

*Variables used in the main analyses; +30 min, cortisol collected 30 min after wakening.

Boys and girls in the sample did not differ in the cortisol levels of the mothers during pregnancy [evening cortisol: t(67.24) = 1.02, p > 0.05; cortisol decline: t(70.42) = 0.84, p > 0.05].

**TABLE 3:** Pearson’s correlations between all prenatal maternal cortisol variables.

		Day 1					Day 2					Day 1 & 2				
				
		Wakening	+30 min	12:00	16:00	Evening	Wakening	+30 min	12:00	16:00	Evening	Wakening	+30 min	12:00	16:00	Evening
Day 1	Wakening	−	−	−	−	−	−	−	−	−	−	−	−	−	−	−
	+ 30 min	0.43**	−	−	−	−	−	−	−	−	−	−	−	−	−	−
	12:00	0.18	0.22	−	−	−	−	−	−	−	−	−	−	−	−	−
	16:00	0.12	0.17	0.54**	−	−	−	−	−	−	−	−	−	−	−	−
	Evening	0.22	0.29*	0.61**	0.65**	−	−	−	−	−	−	−	−	−	−	−
Day 2	Wakening	0.63**	0.45**	0.21	0.08	0.31*	−	−	−	−	−	−	−	−	−	−
	+ 30 min	0.31*	0.49**	0.11	0.28*	0.19	0.36*	−	−	−	−	−	−	−	−	−
	12:00	0.10	–0.03	0.54**	0.55**	0.52**	0.20	0.22	−	−	−	−	−	−	−	−
	16:00	0.10	0.07	0.43**	0.46**	0.44**	0.20	0.33*	0.52**	−	−	−	−	−	−	−
	Evening	0.30*	0.08	0.52**	0.50**	0.55**	0.30*	0.02	0.50**	0.40**	−	−	−	−	−	−
Day 1 & 2	Wakening	0.91**	0.49**	0.18	0.08	0.26*	0.91**	0.42**	0.19	0.18	0.30*	−	−	−	−	−
	+ 30 min	0.40**	0.90**	0.22	0.23	0.24*	0.41**	0.88**	0.06	0.22	0.003	0.47**	−	−	−	−
	12:00	0.21	0.12	0.91**	0.58**	0.64**	0.25	0.17	0.89**	0.49**	0.56**	0.25*	0.17	−	−	−
	16:00	0.13	0.12	0.57**	0.85**	0.62**	0.16	0.37**	0.64**	0.86**	0.51**	0.16	0.25*	0.62**	−	−
	Evening	0.25*	0.18	0.66**	0.66**	0.91**	0.33**	0.16	0.58**	0.46**	0.87**	0.28**	0.15	0.68**	0.65**	−

**p < 0.001; *p < 0.05; +30 min, cortisol collected 30 min after wakening.

### Magnetic resonance imaging parameters

Brain image acquisition was performed using a 3T MAGNETOM PrismaFit MRI scanner (Siemens AG, Healthcare Sector, Erlangen, Germany) with a 32 channel-coil. The children underwent imaging in the supine position. Structural whole-brain T1-weighted images were acquired using a MPRAGE sequence (TR = 2,300 ms, TE = 3.03 ms, 192 sagittal slices, voxel size = 1.0 mm × 1.0 mm × 1.0 mm, FOV = 256 mm × 256 mm). High-resolution structural images with T2 contrast (TR = 8,020 ms, TE = 50 ms, 30 coronal slices, voxel size = 0.4 mm × 0.4 mm × 2.0 mm, FOV = 175mm) were acquired to discern the anatomical details of the hippocampus. The field of view was positioned perpendicular to the long axis of the right hippocampus on a sagittal scout image.

### Magnetic resonance imaging preprocessing and analysis

The pre-registered analysis plan for this study can be found on https://aspredicted.org/qc7ya.pdf. We first conducted a whole-brain multiple regression analysis (Voxel-based morphometry; VBM) with measures of prenatal maternal cortisol as predictor variables and (voxel-wise) gray matter volume as outcome variable. Next, we tested for these effects within *a priori*-specified ROIs, that is, the amygdala and hippocampus. In the second analysis, we performed multivariate multiple regression analysis with extracted hippocampal subfield volumes as outcome variables and prenatal maternal cortisol as predictors. Additional control analyses that were conducted are described here as exploratory. For both the VBM model and hippocampal subfield analyses, these included (1) adding two interaction terms (evening cortisol × gender and cortisol decline × gender); (2) testing aberrant cortisol levels (extremely low and extremely high); and (3) repeating the main analysis with amygdala and hippocampus volumes extracted with FreeSurfer. We conducted a final exploratory VBM analysis with prenatal maternal cortisol awakening response—another indicator of HPA axis functioning.

#### Voxel-based morphometry

Structural T1-weighted images were preprocessed using Statistical Parametric Mapping (SPM12)^1^, which is implemented in MATLAB (version 2019a). The raw T1 images were checked for anatomical abnormalities, movement artifacts, and alignment to the anterior commissure. The Template-O-Matic (TOM; version 1) toolbox was used to create a custom pediatric tissue probability map with age and gender as covariates. The TOM toolbox provides reference data, based on imaging data from the National Institutes of Health (NIH) study of normal brain development for creating customized tissue probability maps. Diffeomorphic Anatomical Registration Through Exponentiated Lie (DARTEL) algorithm was used for tissue segmentation and inter-subject registration of the gray matter images to a group average template image. The customized DARTEL template was used for the normalization of the gray matter images into Montreal Neurological Institute (MNI) space, and the images were smoothed using a 8 mm × 8 mm × 8 mm FWHM Gaussian kernel. Data quality of the normalized and smoothed gray matter maps was checked using the “Check Sample Homogeneity” function of the Computational Anatomy Toolbox 12 (CAT12). Data of six children were indicated as potential outliers—based on a mean correlation of the sample below two standard deviations. After visual inspection, data of two children were excluded from further analysis due to insufficient data quality.

The segmented, normalized, and smoothed gray matter images were entered into a whole-brain multiple regression analysis to investigate regional gray matter density. Standardized prenatal cortisol variables, that is, evening cortisol and cortisol decline, were entered as predictors and age, gender, and total intracranial volume (TIV) as covariates of no interest. Whole-brain statistical inference was assessed using non-parametric permutation tests with the Threshold-Free Cluster Enhancement (TFCE) Toolbox in SPM12 (5,000 permutations). The statistics threshold was set to *p* < 0.05 corrected for family-wise error at a whole-brain level. The TFCE algorithm aims to enhance spatially contiguous signal without being dependent on threshold-based clustering. TFCE values at each voxel represent both spatially distributed cluster size and height information. This approach is especially advantageous for VBM data (Smith and Nichols, 2009; Li et al., 2017). For ROI analysis, we used masks from the AAL Atlas (Tzourio-Mazoyer et al., 2002) for the amygdala and hippocampus.

#### Hippocampus subfield segmentation

Segmentation of the hippocampal subfields was performed using the Automated Segmentation of Hippocampal Subfields (ASHS) tool developed by Yushkevich et al. (2015). This toolbox uses the structural T1-weighted and high-resolution T2-weighted images for each subject as input and provides estimates of hippocampal subfields and neighboring regions. A customized atlas derived from a lifespan sample (Bender et al., 2018) was used to determine hippocampus subfields. For this study, the ASHS Tool was used to extract GMV estimates from the following hippocampal subfields: The dentate gyrus (DG), the subiculum (SUB), the entorhinal cortex (EC), and the cornu ammonis 1 (CA1). Data quality was checked through visual inspection after segmentation. None of the hippocampus scans were indicated as potential outliers, and thus, no data were excluded. The volumes of the extracted hippocampal subfields are presented in Table 4.

**TABLE 4:** Mean volumes of hippocampal subfield volumes (in mm^3^) in boys and girls.

	Boys	Girls
CA1	360.52 ± 102.52	383.87 ± 76.14
EC	433.62 ± 103.23	489.38 ± 71.33
DG	869.45 ± 148.48	917.81 ± 126.33
SUB	965.25 ± 158.67	1048.11 ± 121.46

Values are presented as mean ± SD.

CA1, cornu ammonis 1; EC, entorhinal cortex; DG, dentate gyrus; SUB, subiculum.

We performed a multivariate regression analysis with the bilateral hippocampus subfield volumes (corrected for TIV) as outcome variables and the standardized prenatal cortisol variables (evening cortisol and cortisol decline) as covariates. Age and gender were entered as covariates of no interest. All analyses were performed in R studio software (R version 3.6.1).

## Results

### Voxel-based morphometry results

In the whole-brain VBM analysis, we did not observe any significant associations between the prenatal cortisol measures (i.e., evening cortisol and cortisol decline) and whole-brain GMV. Several exploratory analyses were conducted to further investigate the effects of maternal cortisol on GMV. First, we performed a small volume correction to investigate effects in the amygdala and the hippocampus, regions indicated to be involved in social processing and emotion regulation (Phelps, 2004), and highly susceptible to the influence of stress hormones (Sapolsky et al., 2000). We did not observe any significant relations between the maternal cortisol variables and GMV in these regions. Second, since findings in the literature suggest possible gender differences in the susceptibility to prenatal cortisol, we investigated interactions between gender and the cortisol variables by adding two additional interaction terms to the multiple regression analysis. Neither the evening cortisol × gender, nor the cortisol decline × gender interaction term was significantly associated with whole-brain, amygdala, and hippocampus GMV. To test whether aberrant cortisol levels in both directions (extremely low/extremely high) are related to differences in GMV, we entered a median absolute deviation [MAD: abs(raw score – median)] score for each cortisol variable to the regression model. We did not observe any significant associations between the MAD cortisol scores and whole-brain, amygdala, and hippocampus GMV. Lastly, we repeated the analysis with the CAR measure, but also did not find any significant associations on the whole-brain level or in the amygdala and hippocampus.

To replicate these null findings, we repeated our main analysis with extracted amygdala and hippocampus volumes using FreeSurfer (version 7.1.1.). There were no significant associations between the cortisol variables and the extracted amygdala and hippocampus GMV.

### Hippocampal subfield volumetric analysis results

Maternal prenatal cortisol variables did not significantly predict any of the four hippocampal subfield volumes in the multivariate regression analysis. However, the EC and SUB subfield models were significant [EC: *F*(4, 68) = 3.23, *p* < 0.05; SUB: *F*(4, 68) = 3.30, *p* < 0.05], with a significant main effect of gender. In both subfields, females showed larger volumes compared to males (EC: β = −58.02, *t* = −2.75, *p* < 0.05; SUB: β = −82.08, *t* = −2.47, *p* < 0.05). For the SUB subfield, there was an additional main effect of age, with lower volumes in older children compared to younger children (β = −135.17, *t* = −2.17, *p* < 0.05). There were no significant findings in the CA1 and the DG subfield [CA1: *F*(4, 68) = 1.69, *p* > 0.05; DG: *F*(4, 68) = 1.66, *p* > 0.05], although trend effects were present. Smaller CA1 volumes tended to be related to higher maternal evening cortisol levels (β = −19.25, *t* = −1.7, *p* = 0.09) and larger DG volumes to cortisol decline (β = 31.61, *t* = 1.81, *p* = 0.07). Table 5 summarizes the results of the final regression model.

**TABLE 5:** Results of the multivariate regression analysis by hippocampal subfields.

	β	*t*	*p*	*F*	*df*	*p*	*Adj. R^2^*
CA1							
Overall				1.69	4, 68	0.16	0.04
Cort decline	5.05	0.44	0.66				
Evening cort	–19.25	–1.7	0.09				
Age	54.29	1.35	0.18				
Gender	–27.60	–1.29	0.20				
EC							
Overall				3.23	4, 68	0.02*	0.11
Cort decline	7.50	0.67	0.51				
Evening cort	–17.55	–1.58	0.12				
Age	–45.47	–1.15	0.25				
Gender	–58.02	–2.75	0.01*				
DG							
Overall				1.66	4, 68	0.17	0.04
Cort decline	31.61	1.81	0.07				
Evening cort	–5.60	–0.32	0.75				
Age	8.29	0.14	0.89				
Gender	–43.59	–1.33	0.19				
SUB							
Overall				3.30	4, 68	0.02*	0.11
Cort decline	13.66	0.78	0.44				
Evening cort	–14.08	–0.80	0.43				
Age	–135.17	–2.17	0.03*				
Gender	–82.08	–2.47	0.02*				

Gender was entered as a dummy variable (male = 1, female = 0). The analysis was performed with bilateral subfield volumes.

CA1, cornu ammonis 1; EC, entorhinal cortex; DG, dentate gyrus; SUB, subiculum.

*p < 0.05.

Similar to the whole-brain analysis, we conducted an exploratory analysis by adding the interaction terms evening cortisol x gender and cortisol decline × gender, to the regression analysis. We did not find significant associations between the interaction terms and any of the hippocampal subfield volumes. Furthermore, we entered a median absolute deviation score [MAD; abs(raw score – median)] for each cortisol variable into the regression model to test the effects of aberrant cortisol levels in both directions (extremely low/extremely high) on hippocampal subfield volumes. The MAD scores of the cortisol variables did not predict any of the hippocampal subfield volumes.

Prior to the hippocampal subfield analysis, the diagnostic residual scatterplot of the regression analysis was checked for problems regarding homoscedasticity, linearity, and influential cases. We did not observe any problems regarding these scatterplots. We performed a Shapiro–Wilk test, which indicated that the residuals of the multivariate model were normally distributed (*W* = 0.99394, *p* > 0.05).

## Discussion

In this study, we investigated whether incubated effects of maternal cortisol concentrations during pregnancy manifest in individual variation in brain structure during early adolescence. Based on the previous literature, we expected that aberrant levels of prenatal cortisol are a key physiological mechanism underlying the link between prenatal stress and GMV alterations in the offspring. In line with this assumption, maternal cortisol is thought to influence the fetal brain and later influence offspring neurodevelopment at the onset of puberty. We hypothesized to find associations between individual differences in GMV in typically developing 12-year-old children and maternal prenatal cortisol, using two well-established markers: evening cortisol and cortisol decline. Our findings indicate that variations in maternal cortisol concentrations during late pregnancy are not related to brain structure at the onset of puberty, either at the whole-brain level, in stress-sensitive regions or when focusing on specific subfield volumes of the hippocampus. Null effects in total amygdala and hippocampus volumes were replicated using FreeSurfer software.

Although regions such as the prefrontal cortex, amygdala, and hippocampus have previously been shown to be highly susceptible to stress particularly occurring early in life (Lupien et al., 2009), we do not report similar effects in our sample of 12-year-old adolescents. These null findings are consistent within our study across various levels of specification (whole-brain, region of interest, and individual hippocampal subfields) and analysis software (SPM, FreeSurfer, ASHS) and are based on a comparable sample size to that used in other studies. Only a few studies have examined individual differences in neural structures associated with prenatal stress, and even less with prenatal cortisol, with mixed findings. For example, self-reported prenatal stress was related to larger posterior parietal cortex volumes of adolescents (aged 11–14 years; McQuaid et al., 2019). Higher cortical thickness in frontal regions was related to elevated late-pregnancy maternal plasma cortisol levels (Davis et al., 2017), while in another study, early, but not late, higher prenatal maternal cortisol levels were related to enlarged amygdala, but not hippocampus volume (Buss et al., 2012). Due to the limited number of studies and the different age ranges included in the samples, comparing these results should be done cautiously. Important differences among studies with respect to pregnancy period, the type of brain measure used (GMV, cortical thickness), age range, and pubertal developmental stage may underlie inconsistencies between findings. For example, only Buss et al. (2012) specifically investigated GM changes related to (salivary) prenatal maternal cortisol, but did so in a younger sample of children (6–9 years old) than those included in the current study.

Another consideration is the heterogeneity in cortisol assessment methods (blood versus saliva cortisol) between studies, which may lead to inconsistent findings. Typically, free cortisol in saliva is 10–35% lower than in the blood, but because salivary cortisol is highly correlated to unbound free plasma cortisol concentrations, it is possible to compare effects derived from blood and saliva cortisol (Levine et al., 2007). However, during pregnancy, corticosteroid-binding globulin levels rise (Levine et al., 2007) and cause the relationship between total blood concentration and salivary cortisol concentration to become non-linear (Hellhammer et al., 2009). This change during pregnancy may lead to divergent findings among studies using different cortisol assessment methods (e.g., Davis et al., 2017 investigated blood cortisol levels).

Methodological differences *within* saliva cortisol assessments may also contribute to inconsistent findings. In contrast to studies with only a single saliva cortisol sample per time point (Buss et al., 2012), we collected saliva samples five times a day, on two consecutive days and subsequently calculated mean concentrations over these two days. Such a multi-sample approach is essential for a reliable assessment of salivary cortisol, since a single measure has been shown to be only weakly correlated (*r* = 0.1) with the AUCg of several cortisol measurements during the day (Harville et al., 2007).

The hippocampus in particular has been a primary target in prenatal stress research given its high density of glucocorticoid receptors (Sapolsky et al., 2000) and its role in a variety of psychological disorders (Sala et al., 2004). Within the hippocampus, the dentate gyrus and CA subfields have been identified as especially susceptible to stress effects (McEwen Harold and Milliken, 2000). Hence, and to overcome the caveat of treating the hippocampus as a single structural entity (total volume), we explicitly segmented the hippocampus to investigate stress-induced effects in different subfields. In our sample, maternal prenatal cortisol was not associated with either total hippocampal volume or individual hippocampal subfield volumes (DG, CA1, EC, and SUB; although evening cortisol marginally predicted smaller CA1 volume and cortisol decline marginally predicted larger DG volume). As such, our findings are partially in line with Buss et al. (2012), who only report alterations in amygdala but not hippocampal volume in relation to (salivary) prenatal maternal cortisol levels. In general, early life stress-related hippocampal GM changes in adolescents remain inconsistent with a number of studies reporting null effects (De Bellis et al., 2001; Tupler and De Bellis, 2006; Tottenham et al., 2010). It has, therefore, been suggested that stress-induced changes in hippocampal volume may be difficult to observe during development, but manifest later in life (Tottenham and Sheridan, 2010). Perhaps also in our sample, individual developmental differences at the onset of puberty obscured early stress effects, particularly in the hippocampus, but also in other structures undergoing maturational restructuring (such as the prefrontal cortex). Repeated assessment of these individuals could therefore potentially reveal incubated effects at a later point in time. Alternatively, early life stress, and particularly prenatal stress, could be related to offspring neurodevelopment outcomes only at a young age, before puberty-related changes take place.

Even though we could not find neurodevelopmental associations with prenatal cortisol, previous work using the same dataset has linked prenatal cortisol to various child outcomes. Higher levels in maternal evening cortisol during pregnancy predicted more health problems in the child during the first year of life (Beijers et al., 2010) and a higher physiological stress response of the child at 6 years of age (Simons et al., 2019). Also, in the same sample, higher scores of prenatal cortisol decline were related to more child digestive illnesses till 3.5 years of age (Zijlmans et al., 2017), and a combination of high reported stress and high cortisol concentrations during pregnancy altered infant microbiota composition (Zijlmans et al., 2015). Prenatal cortisol could play a greater role in predicting child physiology and health rather than individual differences in offspring neurodevelopment, 12 years later.

Although the “absence of evidence” is not “evidence of absence,” we also have to acknowledge that a possible explanation for null effects in our study may be a more moderate role of prenatal maternal cortisol, as opposed to what is suggested in the literature. Aberrant levels of maternal cortisol that occur during pregnancy are thought to constitute the basis of an important mechanism by which prenatal maternal stress leads to compromised offspring outcomes. However, several studies have reported correlations between prenatal cortisol and prenatal stress as low or non-significant (e.g., Beijers et al., 2010; Davis and Sandman, 2010, 2012; de Weerth et al., 2013). This is corroborated by a systematic review conducted by Zijlmans et al. (2015) showing that only about a quarter of the analyses of studies that had investigated associations between prenatal cortisol and child outcomes actually found significant relationships between them. These studies suggest that cortisol is not the sole physiological marker underlying the relationship between maternal stress and child development (Beijers et al., 2014; de Weerth, 2018). Recent studies show that placental CRH and the maternal immune system, in particular interleukin-6 concentrations, may also function as possible biological pathways of influence (Sandman et al., 2018; Rasmussen et al., 2019).

Despite several strengths of the study, including a prospective longitudinal design from pregnancy until the onset of puberty (age 12 years), the use of well-established markers of prenatal cortisol, and investigation of specific hippocampal subfield volumes, there are some limitations that should be considered. The BIBO study participants could be considered a relatively selective sample with respect to socioeconomic status (e.g., 76% of mothers had a college or university education level), which tended to be more diverse in other studies (see Beijers et al., 2010 for BIBO demographic details). As such, the mothers in this study might have been less likely to experience adverse and stressful events during their pregnancy, which would have impacted alternations in their cortisol concentrations. While the sample size is sufficient for finding a medium effect size, perhaps in this healthy sample with few prenatal stressful events, the effect of prenatal maternal cortisol may in fact be much smaller than anticipated. In addition to this, maternal cortisol was measured only during late pregnancy, a period characterized by greatly increased cortisol concentrations (de Weerth and Buitelaar, 2005b). Cortisol samples collected in the first half of pregnancy, because of their naturally lower concentrations, may provide more opportunities for detecting relevant individual differences in maternal cortisol. Because only a single time point was assessed in this study, the results should be interpreted with caution with respect to prenatal maternal cortisol effects spanning a wider gestational period. Finally, it would be important to consider other potential intervening factors, such as child exposure to negative life experiences or parental mental health problems, in future analyses linking prenatal maternal cortisol and child outcomes.

By repeatedly assessing cortisol during various time points during pregnancy, future studies could investigate a more precise timing of cortisol exposure, for example, with growth curve analysis over the gestational period. Other informative markers that could be included in future studies are hair and nail cortisol, which would enable measuring chronic stress experienced by the mother and fetal exposure to cortisol over the course of the pregnancy (Meyer and Novak, 2021).

## Conclusion

Prenatal maternal cortisol concentrations were not associated with individual differences in offspring brain structure at the onset of puberty—either on the whole-brain level, in stress-sensitive regions, or in specific hippocampal subfield volumes. The results of this imply that the role of maternal stress hormones on offspring neurodevelopment may not be as robust as previously reported for child physiology and health or that these associations are no longer apparent at the onset of puberty or, in contrast, become apparent later in development. It is important to bear in mind that these interpretations are limited with respect to cortisol in the late gestational period. By including several measures of cortisol throughout the course of gestation, future research could disambiguate what role timing of exposure plays on offspring structural brain development. Finally, future studies should also investigate other potential mechanisms underlying the link between maternal prenatal stress and child neurodevelopment and health outcomes.

## Data availability statement

The datasets presented in this article are not readily available because the dataset on which these analyses are conducted belong to the historical longitudinal BIBO (Basale Invloeden op de Baby Ontwikkeling) study, which started in 2006. In accordance with the informed consent approved by the Ethical Committee of the Faculty of Social Sciences, Radboud University, Nijmegen (ECG300107/SW2017-1303-497/SW2017-1303-498) and signed by the participants, data cannot be shared in public repositories. However, access to the data can be requested to the data access manager Irene van Kroonenburg (irene.vankroonenburg@radboudumc.nl).

## Ethics statement

The studies involving human participants were reviewed and approved by (1) the Local Ethical Committe Commissie Mensgebonden Onderzoek (CMO) Region Arnhem – Nijmegen; (2) Ethical Committee of the Faculty of Social Sciences of Radboud University. Written informed consent to participate in this study was provided by the children and the participants’ legal guardian/next of kin.

## Author contributions

AT, RB, KG, SK, CdW, and KR: conceptualization. AT, KG, RB, SK, and CdW: methodology. AT, KG, and SK: formal analysis. AT and KG: investigation and writing—original draft. AT, RB, and CdW: data curation. AT, RB, SK, CdW, and KR: supervision and writing—reviewing and editing. All authors contributed to the article and approved the submitted version.

## Footnotes

1
www.fil.ion.ucl.uk/spm
